# Symptomatik einer akuten SARS-CoV-2-Infektion bei Kindern im Kita-Alter

**DOI:** 10.1007/s00112-022-01640-3

**Published:** 2022-11-09

**Authors:** Juliane Wurm, Ann-Sophie Lehfeld, Gianni Varnaccia, Helena Iwanowski, Bianca Finkel, Anja Schienkiewitz, Hanna Perlitz, Anne-Kathrin Mareike Loer, Barbara Wess, Andrea Franke, Antje Hüther, Tim Kuttig, Anna Sandoni, Ulrike Kubisch, Susanne Jordan, Walter Haas, Udo Buchholz, Julika Loss

**Affiliations:** 1 Abteilung für Epidemiologie und Gesundheitsmonitoring, Robert Koch-Institut, General-Pape-Straße 62–66, 12101 Berlin, Deutschland; 2 Abteilung für Infektionsepidemiologie, Robert Koch-Institut, Berlin, Deutschland

**Keywords:** Ausbruchsuntersuchung, Meldedaten, Corona-KiTa-Studie, Schnupfen, COVID-19, Outbreak invesitgations, Surveillance data, Corona-KiTa study, Rhinitis, COVID-19

## Abstract

**Hintergrund:**

Die Symptomatik einer pädiatrischen SARS-CoV-2-Infektion ist sehr variabel. Es gibt nur wenige Studien zu nichthospitalisierten Kindern bzw. Kindern im Kita-Alter.

**Ziel der Arbeit:**

Die Arbeit soll die Häufigkeit verschiedener COVID-19-Symptome bei ein- bis 6‑jährigen Kindern beschreiben. Sie führt dazu Daten aus 2 Modulen der Corona-KiTa-Studie zusammen: 1) das Modul „COALA“ – Corona: Anlassbezogene Untersuchungen in Kitas und 2) das Modul „CATS“ – Corona KiTa Surveillance (Meldedaten).

**Material und Methoden:**

In COALA wurden die Infektionsgeschehen in 30 Kitas, in denen ein SARS-CoV-2-Fall gemeldet wurde, untersucht (Oktober 2020 bis Juni 2021). Kita-Kinder wurden prospektiv über 12 Tage beobachtet (SARS-CoV-2-Tests, Symptomtagebuch). Die Ergebnisse wurden mit den Symptomangaben der deutschlandweit gemeldeten SARS-CoV-2-Fälle (Meldedaten) verglichen.

**Ergebnisse:**

Aus den teilnehmenden Kitas liegen für 289 Kinder Angaben vor. Von 39 Kindern mit SARS-CoV‑2 (Wildtyp, α‑Variante) hatten 64 % mindestens ein Symptom, von den nicht mit SARS-CoV‑2 infizierten Kindern 40 %. In beiden Gruppen war Schnupfen das häufigste Symptom (36 % vs. 25 %, n. s.). Aus den Meldedaten liegen für 84.371 Kinder klinische Informationen vor, Fieber war am häufigsten (27 %), neben Schnupfen (26 %). Schwere Beschwerden wie z. B. Atemnot wurden in den Ausbruchsuntersuchungen und in den Meldedaten nur selten angegeben (3 % bzw. 1 %).

**Schlussfolgerung:**

Kinder im Kita-Alter haben meist milde bzw. asymptomatische Verläufe einer SARS-CoV-2-Infektion. Ihre Symptome ähneln denjenigen von nicht mit SARS-CoV‑2 infizierten Kindern aus denselben Kitas. Es erscheint sinnvoll, Erkenntnisse aus den Meldedaten durch Ausbruchsuntersuchungen zu ergänzen, um methodische Limitationen der einzelnen Vorgehensweisen auszugleichen.

**Zusatzmaterial online:**

Die Online-Version dieses Beitrags (10.1007/s00112-022-01640-3) enthält eine weitere Tabelle, die Symptome bei symptomatischen SARS-CoV-2-Fällen von Kindern im Alter von einem bis 6 Jahren in der COALA-Stichprobe und in den Meldedaten gegenüberstellt.

## Abstract (en)

**Background:**

The symptoms of SARS-CoV‑2 infections in children are mostly mild; however, the symptoms are highly variable. There are only a few studies on non-hospitalized children. The clinical picture described in hospitalized children cannot be transferred to non-hospitalized children and the frequency of certain symptoms in children may thus be overestimated. Furthermore, most studies include a broad age group (up to 18 years). The symptoms of younger children have so far been described in less detail.

**Objective:**

The paper aims to describe the frequency of COVID-19 symptoms in younger children (1–6 years old). Data of the two modules COALA (*Corona: Anlassbezogene Untersuchungen in Kitas*) and CATS (Corona-KiTa surveillance) of the Corona-KiTa study are evaluated and the results of the two studies are compared and discussed against the background of the different methodologies. In the COALA study, the type and frequency of symptoms of children infected with SARS-CoV‑2 are evaluated and compared to symptoms of children who tested negative for SARS-CoV‑2. Symptom frequencies of SARS-CoV‑2 infected children of the COALA study are compared with data collected from surveillance data (CATS).

**Material and methods:**

The COALA study investigated 30 SARS-CoV‑2 outbreaks in day care centers where at least 1 SARS-CoV‑2 case was reported between October 2020 and June 2021. Using a prospective study design, day care children who were infected with SARS-CoV‑2 and their contact persons were studied over a period of 12 days (including regular SARS-CoV‑2 testing, retrospective interviews and daily symptom reporting). The results from the COALA study were compared with data from COVID-19 surveillance cases (CATS) for the same age group and time period. In Germany, SARS-CoV‑2 cases are reported to the local health authorities by physicians and laboratories. When reporting cases symptoms can be reported as well.

**Results:**

From the COALA study, interview and reported symptom data were available for 289 children from the participating day care centers. Of 39 children with a SARS-CoV‑2 infection (wild-type, α‑variant), 64% had at least 1 symptom; of the children who tested negative for SARS-CoV‑2, 40% had at least 1 symptom. In both groups, rhinitis was the most common symptom (36% vs. 25%, n. s.). From the surveillance data (CATS), clinical information was available for 84,371 SARS-CoV‑2 positive children; fever was most common (27%) along with rhinitis (26%). Severe symptoms such as dyspnea were rarely reported in the outbreak investigations and in the surveillance data (3% and 1%, respectively).

**Conclusion:**

Day care-aged children infected with SARS-CoV‑2 usually have mild or asymptomatic courses. Their symptoms are similar to those of children who tested negative for SARS-CoV‑2 from the same day care centers; thus, the observed COVID-19 symptoms are nonspecific. Combining data from the two modules is useful: findings from a very large database, as provided by the surveillance data, are complemented by findings from day care center outbreaks, where detailed prospective data on infected children can be compared with those of children who tested negative for SARS-CoV‑2.

## Hintergrund und Fragestellung

Seit Beginn der „Coronavirus-disease-2019“(COVID-19)-Pandemie sind Kinder in allen Altersgruppen von Infektionen mit dem „severe acute respiratory syndrome coronavirus type 2“ (SARS-CoV‑2) betroffen. Das klinische Bild ist dabei hoch variabel, und die Art und Schwere der Symptome unterscheiden sich zwischen Altersgruppen [4]. Mittlerweile gibt es mehrere internationale Studien und Übersichtsarbeiten, die die Symptomatik von Kindern auswerten [2, 5, 8]. Sie zeigen, dass eine SARS-CoV-2-Infektion bei Kindern überwiegend mild verläuft. Ein großer Teil der infizierten Kinder bleibt asymptomatisch. Schwere oder letale Krankheitsverläufe sind selten. Die Art der Symptome ist unterschiedlich; häufig berichtete Symptome bei Kindern sind Fieber, Kopfschmerzen und Husten. Zahlreiche Studien zur Symptomatik infizierter Kinder basieren auf Daten von hospitalisierten Kindern bzw. von Kindern, die ärztlich vorstellig wurden. Das beschriebene Krankheitsbild lässt sich nicht ohne Weiteres auf nichthospitalisierte Kinder übertragen, und die Häufigkeit bestimmter Symptome bei Kindern wird dadurch möglicherweise überschätzt. Weiterhin schließen die Studien zumeist eine breite Altersgruppe (bis 18 Jahre) ein. Die Symptomatik jüngerer Kinder (im Kita-Alter) ist bisher weniger detailliert beschrieben.

In der Corona-KiTa-Studie wurden die Symptome von Kindern im Kita-Alter, die mit SARS-CoV‑2 infiziert waren, erfasst. Grundlage dieser interdisziplinären Studie sind zum einen Primärdaten, die bei Kindern, die anlässlich eines akuten SARS-CoV-2-Infektionsgeschehens in Kitas rekrutiert worden waren (Modul „COALA“, Corona: Anlassbezogene Untersuchungen in Kitas) erhoben wurden, zum anderen die nach Alter stratifizierten Meldedaten der Gesundheitsämter (Modul „CATS“, Corona KiTa Surveillance). Die Daten wurden ausgewertet, um folgende Fragen zu beantworten:

- Welche Symptome haben Kinder im Kita-Alter (eins bis 6 Jahre), die mit SARS-CoV‑2 infiziert sind, und mit welcher Häufigkeit treten verschiedene Symptome auf?
- Welche Unterschiede zeigen sich zu klinischen Beschwerden nicht mit SARS-CoV‑2 infizierter kindlicher Kontaktpersonen?
- Wie unterscheiden sich Symptomhäufigkeiten in den Meldedaten von denen im Rahmen einer Ausbruchsuntersuchung erhobenen Daten?

## Studiendesign und Untersuchungsmethoden

### Studiendesign der COALA-Studie, Erfassung und Auswertung der Symptomatik

Zwischen Oktober 2020 und Juni 2021 wurde das Infektionsgeschehen in 30 Kitas, in denen ein SARS-CoV-2-Fall gemeldet wurde, untersucht. Mit einem prospektiven Studiendesign wurden mit SARS-CoV‑2 infizierte Kita-Kinder sowie deren exponierte Kontaktpersonen in der Kitagruppe und in den Haushalten untersucht. Vier bis sechs Tage nach dem Testergebnis des Indexfalls wurden Hausbesuche bei den Teilnehmenden durchgeführt. Es wurden kombinierte Mund-Nasen-Abstriche durchgeführt und Speichelproben gewonnen, die per Real-time-Reverse-Transkriptase-Polymerase-Kettenreaktion (rRT-PCR) auf SARS-CoV‑2 getestet wurden. Alle Teilnehmenden wurden anschließend über einen Zeitraum von 12 Tagen beobachtet. In den Hausbesuchen wurden die Erwachsenen angeleitet, ihre Kinder regelmäßig zu beproben und das Auftreten von Symptomen anhand von standardisierten Symptomtagebüchern täglich zu dokumentieren. Es konnten die Symptome Fieber, Schüttelfrost, anhaltender Husten, Atemnot/Kurzatmigkeit, Schmerzen beim Atmen, Kopfschmerzen, Gliederschmerzen, Übelkeit, Durchfall, Riech‑/Geschmacksstörung oder andere Symptome (Freitext) ausgewählt werden. Anhand der Symptomtagebücher wird die Symptomatik beschrieben und die Symptomatik der positiv und negativ (Kontrollgruppe) auf SARS-CoV‑2 getesteten Kinder verglichen. Zudem wurden die möglicherweise vor dem Hausbesuch aufgetretenen Symptome in standardisierten Telefon-Interviews retrospektiv erfasst. Die Interviews wurden mit erwachsenen Haushaltsmitgliedern durchgeführt, die die Fragen für ihre Kinder beantworteten. Es wurden alle ein- bis 6‑jährigen Kinder, von denen mindestens ein PCR-Ergebnis, das Symptomtagebuch und Befragungsdaten des Telefoninterviews vorlagen, in die Auswertung eingeschlossen. Als symptomatisch galten Kinder, bei denen mindestens eines der oben genannten Symptome an mindestens einem Tag vorkam. Ausgeschlossen wurden retrospektive Symptomangaben von Sekundärfällen und Kontrollen, die zeitlich vor der Testung des Indexfalles lagen, da angenommen wurde, dass diese nicht im Zusammenhang mit der SARS-CoV-2-Infektion im Rahmen des Kita-Ausbruches standen. Es wurden sowohl für die Kinder mit positivem SARS-CoV-2-Testergebnis als auch für die mit negativem Ergebnis die Häufigkeiten einzelner Symptome ausgewertet. Eine detaillierte Beschreibung des Studiendesigns findet sich bei Schienkiewitz et al. [14].

### Meldedaten: Erfassung und Auswertung der Symptomatik (Modul CATS)

Im Rahmen der Meldepflicht gemäß Infektionsschutzgesetz sind der Verdacht auf eine Erkrankung, eine Erkrankung und Tod in Bezug auf COVID-19 sowie der Nachweis des Erregers SARS-CoV‑2 meldepflichtig [12]. Die Meldung an das Gesundheitsamt erfolgt dabei durch die Ärztinnen und Ärzte sowie durch das Labor, welches die SARS-CoV-2-Infektion nachweist. COVID-19-Fälle, die die Referenzdefinition des Robert Koch-Instituts (RKI) erfüllen (Nachweis von SARS-CoV‑2 mittels Nukleinsäurenachweis oder Erregerisolierung, unabhängig vom klinischen Bild) [12], werden von den Gesundheitsämtern an die zuständige Landesbehörde und von dort an das RKI übermittelt. Dabei haben die Gesundheitsämter in der Meldesoftware die Möglichkeit anzugeben, ob Symptome bei den Betroffenen vorliegen. In einigen Fällen sind diese Informationen jedoch nicht vollständig, weil sie zum Zeitpunkt der Meldung noch nicht vorliegen, nicht Inhalt der Meldung sind und von den Gesundheitsämtern erst ermittelt werden müssen. Bei vorliegenden Informationen zur Symptomatik können mehrere Symptome bzw. klinische Zustände ausgewählt werden, darunter z. B. Fieber, Husten, Schnupfen, Geruchs- und Geschmacksstörungen, Pneumonie und Lungenversagen. Für einen Teil der übermittelten Fälle lagen keine klinischen Informationen vor; dabei kann es sich sowohl um asymptomatische Fälle als auch um Fälle handeln, bei denen die Symptome nicht erhoben wurden, z. B. weil der Patient oder die Patientin bzw. bei Kindern eine sorgeberechtigte Person nicht kontaktiert werden konnte. Ebenfalls ist zu beachten, dass es vorkommen kann, dass zunächst mild erkrankte Personen erst im weiteren Verlauf schwerwiegendere Symptome entwickeln können, diese Information zum Verlauf aber den Gesundheitsämtern nicht immer vorliegt. Um eine Vergleichbarkeit zur COALA-Studie herzustellen, wurden die Symptomangaben der laborbestätigten COVID-19-Fälle bei Kindern im Alter von einem bis 6 Jahren für den Zeitraum von Oktober 2020 (Woche 44) bis Juni 2021 (Woche 23) betrachtet (Datenstand: 09.03.2022).

Tab. 1 stellt die unterschiedlichen Vorgehensweisen beider Studienmodule bei der Erfassung der Symptome gegenüber.

**Table 1:** 

	Ausbruchsuntersuchung (COALA)	Meldedaten (CATS)
Wer wurde untersucht?	Alter: ein bis 6 Jahre	Alter: ein bis 6 Jahre
	SARS-CoV-2-Fälle und Kontrollen (wiederholt negativ getestete Kontaktpersonen)	Nur SARS-CoV-2-Fälle
	Aus 30 SARS-CoV-2-Ausbrüchen in Kitas bundesweit	Aus der Vollerhebung aller bundesweit übermittelten SARS-CoV-2-Fälle
	*Gesamt: n* *=* *334* Mit Angaben zu Symptomen: *n* = 289 39 SARS-CoV-2-Fälle und 250 Kontrollen	*Gesamt: n* *=* *120.215* Mit Angaben zu Symptomen: *n* = 84.371^a^
Zeitraum der Datenerhebung	Oktober 2020 bis Juni 2021	Oktober 2020 bis Juni 2021
Studiendesign/Datenbasis	Anlassbezogene Untersuchung in nichtrepräsentativ gewählten Kitas	Querschnittserhebung, Vollerhebung
	Prospektives Studiendesign, kontinuierliche Erfassung der Symptome über 12 Tage	Retrospektive Erfassung der Symptome
	Zusätzlich retrospektive Erfassung der Symptome	
	Kontrolle: Vergleich mit kindlichen Kontaktpersonen ohne SARS-CoV-2-Nachweis	
	Angabe zu Symptomen über Eltern	
Symptom-Erhebung	*Instrument*	*Instrument*
	Prospektiv: standardisierte Symptomtagebücher (12 Tage)	„Standardisierte“ Abfrage bei SARS-CoV-2-Fall-Meldung
	Retrospektiv: standardisierte Interviews (3 Wochen)	
	*Angaben durch Eltern*	*Übermittlung der Angaben durch Gesundheitsamt*
Mögliche Verzerrung	Eltern von schwer erkrankten Kindern lehnen Studienteilnahme evtl. eher ab oder aber nehmen eher teil, um ein Testergebnis zu erhalten	*Surveillance-Bias* Symptomatische Kinder werden eher auf SARS-CoV‑2 getestet und gemeldet Evtl. werden Kinder mit einer **bestimmten** Symptomatik eher auf SARS-CoV‑2 getestet
	Indexfälle: mögliche Überrepräsentation symptomatischer Fälle	
	Sekundärfälle: geringe Verzerrung, Repräsentativität möglicherweise durch Teilnahmerate (60 %) eingeschränkt	

^a^Im Erhebungszeitraum der Ausbruchsuntersuchungen der COALA-Studie

### Statistische Berechnungen

Es wurden univariate Analysen zur Berechnung der Anteile symptomatischer und asymptomatischer Kinder und des Vorkommens einzelner Symptome durchgeführt. Zur Überprüfung des Zusammenhangs einzelner Symptome mit einem positiven PCR-Test wurden „odds ratios“ berechnet. Alle Analysen erfolgten mittels der Statistik-Software Stata Version 17.0.

## Ergebnisse

### Ergebnisse aus COALA

**Figure 1:**
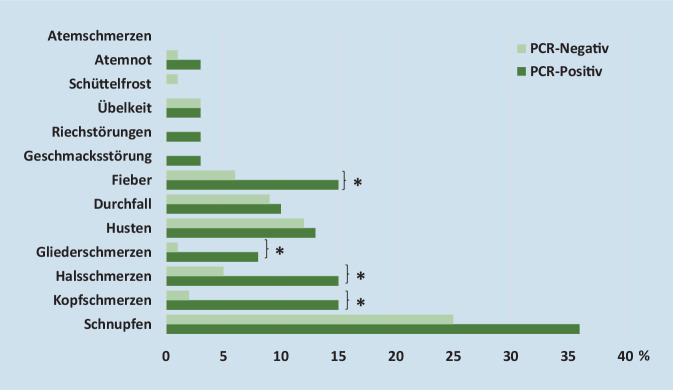

Es wurden 334 Kinder (ein bis 6 Jahre) in die COALA-Studie eingeschlossen. Für 289 Kinder liegen Angaben aus der standardisierten retrospektiven Befragung und dem Symptomtagebuch vor, davon 137 Ein- bis 3‑Jährige und 152 4‑ bis 6‑Jährige (Tab. 2). Darunter befanden sich 39 Kinder mit einem aktuellen SARS-CoV-2-Nachweis. Bei 29 Kindern wurde eine Genomsequenzierung der Virus-RNA vorgenommen, die bei 25/29 Kindern die Alpha-Variante ergab, bei 4/29 Kindern den Virus-Wildtyp. 12/39 positiv getestete Kinder waren die Indexfälle des Ausbruchsgeschehens, 27/39 waren Sekundärfälle innerhalb der Kita (25/27) bzw. in den Haushalten (2/27). Bei 64 % (*n* = 25) der infizierten Ein- bis 6‑Jährigen lagen retrospektiv oder im Tagebuch Symptome vor. Es zeigten sich Altersgruppenunterschiede: Die infizierten Ein- bis 3‑Jährigen waren seltener symptomatisch als die 4‑ bis 6‑Jährigen (36 % vs. 75 %, *p*-Wert 0,024). Insgesamt wiesen die infizierten Kinder durchschnittlich 1,9 verschiedene Symptome auf. Betrachtet man die 27 Sekundärfälle der Kita-Ausbrüche, wiesen 56 % Symptome auf. Auch in dieser Gruppe waren die Ein- bis 3‑Jährigen deutlich seltener symptomatisch als die der 4‑ bis 6‑Jährigen (13 % vs. 74 %, *p*-Wert 0,008). Von den 12 Indexfällen waren 83 % symptomatisch. Das häufigste Symptom der positiv getesteten Kinder war Schnupfen, gefolgt von Kopfschmerzen, Halsschmerzen und Fieber (Abb. 1 und Tab. 3). Riech- und Geschmacksstörungen wurden bei Kindern sehr selten dokumentiert. Für einen großen Teil der negativ auf SARS-CoV‑2 getesteten Kinder (Kontaktpersonen aus der vom Ausbruch betroffenen Kitagruppe/Geschwisterkinder aus den Haushalten) wurden im Beobachtungszeitraum Symptome angegeben (40 %, *n* = 101). Schnupfen war das häufigste Symptom und trat bei mehr als jedem vierten negativ getesteten Kind im zeitlichen Zusammenhang zum SARS-CoV-2-Infektionsgeschehen in der Kita auf. Kopfschmerzen, Halsschmerzen, Fieber und Gliederschmerzen traten bei den SARS-CoV‑2 infizierten Kindern signifikant häufiger auf als bei nicht mit SARS-CoV‑2 infizierten Kindern (*p* < 0,05). Insbesondere das Auftreten von Kopfschmerzen war auffällig: Es war mit einer 9fach erhöhten Chance für einen positiven PCR-Test assoziiert (OR 9,4, *p*-Wert 0,002).

**Table 2:** 

	Indexfall (Fallzahl, *n*)	Sekundärfall (Fallzahl, *n*)	Kontrolle (Fallzahl, *n*)	Gesamt (Fallzahl, *n*)
*Geschlecht*				
Weiblich	5	15	133	137
Männlich	7	12	117	152
*Alter*				
Ein bis 3 Jahre	3	8	126	137
4 bis 6 Jahre	9	19	124	152
*Symptome*				
Symptomatisch	10	15	101	126
Asymptomatisch	2	12	149	163
*Virusvariante*				
Wildtyp	3	1	–	4
Alpha-Variante	5	20	–	25
Kein Sequenzierungsergebnis	4	6	–	10
Nicht zutreffend	–	–	250	250
*Gesamt*	12	27	250	289

**Table 3:** 

Symptom	Ausbruchsuntersuchungen^a^ Fallzahl (*n*) Anteil (in Prozent)	Meldedaten^a^ Fallzahl (*n*) Anteil (in Prozent)
Mindestens ein Symptom	25 64 %	54.382 64 %
Schnupfen	14 36 %	22.195 26 %
Kopfschmerzen	6 15 %	x
Halsschmerzen	6 15 %	6427 8 %
Fieber	6 15 %	22.931 27 %
Husten	5 13 %	22.236 26 %
Durchfall	4 10 %	3.180 4 %
Gliederschmerzen	3 8 %	x
Geschmacksstörung/Geschmacksverlust	1 3 %	958 1 %
Geruchsstörung/Geruchsverlust	1 3 %	718 1 %
Übelkeit	1 3 %	x
Schüttelfrost	0 0 %	x
Atemnot	1 3 %	552 1 %
Atemschmerzen	0 0 %	x
Allgemeine Krankheitszeichen	x	18.196 22 %
Sonstige^b^	– –	264 0,3 %

^a^Symptomnennung unter positiven Kindern; *x* diese Symptome wurden so nicht erfasst

^b^Im Meldesystem konnten außerdem Pneumonie: 78 (0,1 %), ARDS: 93 (0,1 %), Beatmung: 9 (<0,0 %), Tachykardie: 27 (<0,0 %) und Tachypnoe: 57 (0,1 %) erfasst werden, die hier als „Sonstige“ zusammengefasst wurden

### Ergebnisse aus den Meldedaten

Von Oktober 2020 bis Juni 2021 lagen für 84.371 (70 %) der insgesamt 120.215 übermittelten SARS-CoV-2-Fälle unter Kindern (ein bis 6 Jahre) auch klinische Informationen und damit Angaben zur Symptomatik bzw. zum Fehlen von Symptomen vor. Unter den Fällen, für die klinische Informationen übermittelt wurden, wurden bei 64 % (*n* = 54.382) COVID-19 relevante Symptome angegeben (symptomatische Fälle). Bei 26 % (*n* = 22.321) aller Fälle wurde nur ein einzelnes Symptom genannt, v. a. Fieber und Schnupfen (8 % bzw. 7 %) genannt. Insgesamt wiesen die infizierten Kinder durchschnittlich 1,2 verschiedene Symptome auf. Bei den Ein- bis 3‑Jährigen wurden häufiger Beschwerden angegeben als bei den 4‑ bis 6‑Jährigen (70 % vs. 60 %, *p* < 0,001), darunter v. a. Fieber (34 % vs. 22 %), Husten (32 % vs. 22 %) und Schnupfen (30 % vs. 23 %). Äußerst selten wurden in den Meldedaten Geschmacks- und Geruchsstörungen (jeweils 1 %) genannt, ebenso die schwerwiegenden klinischen Symptome wie akutes Lungenversagen (ARDS) oder Beatmungspflicht (< 1 %).

Tab. 3 stellt die Symptomhäufigkeiten in der COALA-Stichprobe und in den Meldedaten gegenüber. In Tab. 4 (Zusatzmaterial online) werden die Symptomhäufigkeiten, bezogen auf die symptomatischen Kinder, gegenübergestellt.

## Diskussion

Kinder im Kita-Alter (ein bis 6 Jahre), die mit SARS-CoV‑2 infiziert sind, haben meist oligo- oder asymptomatische klinische Verläufe. Sowohl in den Meldedaten als auch in der Ausbruchsuntersuchung wurde für 64 % der betroffenen Kinder mindestens ein Symptom angegeben. Dabei war Schnupfen eine der am häufigsten genannten klinischen Beschwerden; er wurde in beiden Modulen für etwa ein Drittel bzw. Viertel der SARS-CoV-2-Fälle angegeben. In der COALA-Studie zeigte sich allerdings, dass Schnupfen – wie auch z. B. Husten, Durchfall oder Übelkeit – interessanterweise nicht häufiger auftrat als bei negativ getesteten Kontrollen in den Kita-Gruppen zum Zeitpunkt des SARS-CoV-2-Ausbruchs. Infizierte Kita-Kinder hatten v. a. deutlich häufiger Kopfschmerzen, im Vergleich zu Kita-Kindern, die sich nicht mit SARS-CoV‑2 infizierten (15 % vs. 2 %). Kopfschmerzen werden in den Meldedaten allerdings nicht als Einzelsymptom erfasst. Bei Berichten über Symptomhäufigkeiten ist auf den jeweiligen methodischen Zugang zu achten, da unterschiedliche Arten der Erfassung oder z. B. eine diagnostische Erwartung bestimmter Symptome bei SARS-CoV‑2 infizierten Kindern in divergierenden Symptomprävalenzen resultieren können. So zeigte sich, dass Symptome wie Fieber und Husten in den Meldedaten deutlich häufiger genannt wurden als in der Stichprobe der COALA-Studie, während Schnupfen in der COALA-Studie häufiger angegeben wurde.

### Vergleich mit anderen Studien

Eine in Brasilien durchgeführte Studie, die wie das COALA-Modul das Übertragungsgeschehen unter Kindern und Jugendlichen untersuchte, zeigte ein ähnliches Ergebnis: Zwar hatten mit 15 % weniger positiv getestete Kinder Symptome, allerdings berichtete auch ein großer Teil (44 %) der Kinder ohne Nachweis einer SARS-CoV-2-Infektion über Symptome [1]. In dieser Studie war ebenfalls eine verstopfte Nase das häufigste Symptom (63 %), gefolgt von Kopfschmerzen (55 %) und Husten (51 %). Allerdings wurden in dieser Studie ältere Kinder und eine breitere Altersspanne (5 bis 19 Jahre) untersucht. Eine Metaanalyse von Sah et al. kam mit 53 % symptomatischen Kindern (unter Ausschluss von Indexfällen, 0 bis 18 Jahre) zu einer vergleichbaren Symptomprävalenz wie bei den Sekundärfällen in der COALA-Stichprobe mit 56 % [13].

In einem Bericht aus den USA wurden die den Centers for Disease Control and Prevention (CDC) zwischen Januar und Mai 2020 gemeldeten SARS-CoV-2-Fälle ausgewertet. In diesem Zeitraum wurden den CDC 20.458 Infektionen von Ein- bis 9‑Jährigen gemeldet. Für 25 % war ein Symptomstatus bekannt, wobei unter den symptomatischen Kindern Fieber das häufigste Symptom war (46 %), gefolgt von Husten (37 %). Diese Auswertungen sind hinsichtlich ihrer Methodik vergleichbar (insbesondere Zusatzmaterial online: Tab. 4), jedoch wurden andere Altersgruppen stratifiziert, wodurch die Angaben nur bedingt vergleichbar sind. Zudem wird, aufgrund fehlender Daten, davon ausgegangen, dass die gemeldeten Häufigkeiten der Symptome die tatsächliche Prävalenz unterschätzen [15].

Die Symptomatik kann von der Virusvariante abhängen. Infektionen mit den SARS-CoV-2-Varianten Delta oder Omikron, die im späteren Verlauf der Pandemie aufgetreten sind, können zu einem klinischen Bild führen, das sich von dem hier beschriebenen unterscheidet. Bisher liegen nur wenige kinderspezifische Daten zur Symptomatik im Rahmen einer Infektion mit der Delta- oder Omikron-Variante vor. Eine in Großbritannien durchgeführte Studie verglich die Symptome von insgesamt 1400 Kindern im Schulalter, die mit der Alpha- bzw. der Delta-Variante infiziert waren. Dabei zeigte sich, dass sich die Art der Symptome ähnelte, die Symptome bei einer Delta-Infektion jedoch etwas schwerer waren. Einzelne Symptome, wie Kopfschmerzen und Fieber, kamen bei einer Infektion mit der Delta-Variante häufiger vor [11]. Zur Symptomatik im Rahmen einer Infektion mit der Omikron-Variante berichtete eine in Südafrika durchgeführte Studie, die 138 Kinder in ihre Auswertung einschloss, Fieber, Husten und Kurzatmigkeit als die häufigsten Symptome. Allerdings wurden hier lediglich hospitalisierte Kinder mit einer größeren Altersspanne (0 bis 13 Jahre) eingeschlossen [3]. Für Erwachsene ist beschrieben, dass Omikron-Infektionen milder verlaufen als Infektionen mit vorherigen Varianten [9]. Eine bisher als Preprint erschienene Auswertung aus den USA bestätigte dies auch für die Gruppe der Kinder [16]. Mit neu auftretenden und sich der Immunität einer früheren Infektion entziehenden Varianten oder abnehmender Immunität, kann es außerdem zu SARS-CoV-2-Reinfektionen kommen. Studienergebnisse für die Altersgruppe der Kinder liegen für Reinfektionen mit der Alpha- bzw. Delta-Variante vor. Reinfektionen konnten dabei nicht mit einer schwereren Erkrankung in Verbindung gebracht werden [10]. Für die Omikron-Variante liegen für Kinder noch keine Studienergebnisse zur Symptomatik bei einer Reinfektion vor.

### Stärken und Limitationen der Studie

Beide Datenerhebungen haben jeweils eigene Stärken und Schwächen und ergänzen sich. So ist eine Verallgemeinerung der Ergebnisse der Ausbruchsuntersuchung (COALA-Modul) aufgrund der kleinen Fallzahl nicht möglich. Dafür bietet das prospektive Studiendesign des COALA-Moduls den Vorteil, dass auch asymptomatische und milde SARS-CoV-2-Infektionsverläufe erfasst wurden, die insbesondere im frühen Pandemiegeschehen 2020/2021 (mit teils eingeschränkten Testkapazitäten) u. U. unentdeckt geblieben wären. Eine systematische Verzerrung durch den bevorzugten Einschluss symptomatischer oder hospitalisierter Kinder wurde so minimiert. Andererseits ist denkbar, dass Familien mit besonders schwer erkrankten Fällen eher nicht teilgenommen haben. Insbesondere die Gruppe der Sekundärfälle unterliegt vermutlich den geringsten Verzerrungen hinsichtlich des Anteils asymptomatischer Kinder. Bei der Gruppe der Indexfälle ist hingegen denkbar, dass diese getestet wurden, weil sie symptomatisch waren und es so zu einer Überrepräsentation symptomatischer Fälle kommt („reporting bias“). Es muss beachtet werden, dass die Symptome durch eine Befragung der Eltern erfasst wurden. Das bedeutet, dass sowohl durch die Eltern beobachtete Symptome (z. B. Schnupfen, Husten), zum anderen von den Kindern geäußerte, subjektive Symptome (z. B. Halsschmerzen, Kopfschmerzen, Gliederschmerzen, Übelkeit), einflossen. Ein Mehrwert ist der Vergleich von positiv auf SARS-CoV‑2 getesteten Kindern mit der negativ getesteten Vergleichsgruppe; so ließen sich der Anteil symptomatischer Kinder und die Spezifität der einzelnen Symptome besser einordnen. Die Meldedaten hingegen sind durch fehlende und teils ungenauere Symptomangaben limitiert, da sie nicht durch die Eltern regelmäßig dokumentiert, sondern über das Gesundheitsamt erhoben und übermittelt wurden. Im betrachteten Zeitraum fehlten bei etwa 30 % der Fälle in den Meldedaten Angaben zur Symptomatik. Da ärztliches Personal möglicherweise v. a. dann eine SARS-CoV-2-Infektion vermutet hat, wenn eine „schwerere“ Symptomatik vorlag, ist nicht auszuschließen, dass dies ein Grund für die häufigeren Angaben von Symptomen wie z. B. Fieber (27 % vs. 15 %) und Husten (26 % vs. 13 %) in den Meldedaten im Vergleich zu den Ausbruchsuntersuchungen ist („surveillance bias“). Das gilt insbesondere für den Beobachtungszeitraum, in dem Reihentestungen in Einrichtungen weitestgehend noch nicht etabliert waren. Ein Vergleich der Anteile asymptomatischer Kinder zwischen dem COALA-Modul und den Meldedaten ist daher nicht sinnvoll. Es ist jedoch davon auszugehen, dass die Daten zu Symptomhäufigkeiten aus den Ausbruchsuntersuchungen geringeren Verzerrungen unterliegen, weil Ursachen für einen potenziellen Surveillance bias hierbei eine kleinere Rolle spielen. Die besondere Stärke der Auswertungen der Meldedaten ist mit über 80.000 Kinder im Alter von einem bis 6 Jahren die verfügbare Größe der Datenbasis, sodass alle von den Gesundheitsämtern übermittelten SARS-CoV-2-Fälle mit Angaben zur Symptomatik für den ausgewählten Zeitraum hiermit ausgewertet werden konnten. Die Zusammenschau der beiden Module ergibt einen deutlichen Mehrwert: Erkenntnisse aus einer sehr großen Datenbasis, wie sie die Meldedaten liefern, werden durch Erkenntnisse aus Kita-Ausbrüchen ergänzt, bei denen detaillierte prospektive Angaben zu infizierten Kita-Kindern mit denen nicht mit SARS-CoV‑2 infizierter Kita-Kinder verglichen werden können.

## Implikationen für Politik und Praxis

Die Daten der Studie zeigen erfreulicherweise, dass schwere Krankheitsverläufe von COVID-19 bei Kindern im Kita-Alter äußerst selten vorkommen. Nichtsdestotrotz zeigt die Mehrheit der mit SARS-CoV‑2 infizierten ein- bis 6‑jährigen Kinder mindestens ein – in der Regel mildes – Symptom im zeitlichen Zusammenhang mit der Infektion. Es lässt sich aber schwer beurteilen, inwieweit die beobachteten Beschwerden tatsächlich von SARS-CoV‑2 verursacht werden oder aber mit einer anderen Infektion zusammenhängen, die zeitgleich in der Kita der betroffenen Kinder kursiert. Die Daten belegen, dass das Vorhandensein von Schnupfen bei Kita-Kindern selbst in Zeiten ausgeprägter Hygiene- und Infektionsschutzmaßnahmen in der Kita ein sehr unspezifisches Symptom ist, da mit SARS-CoV‑2 infizierte und nichtinfizierte Kinder in den Kita-Gruppen ähnlich häufig einen Schnupfen angaben. Kopfschmerzen bei Ein- bis 6‑Jährigen scheinen ein Symptom zu sein, das mit höherer Wahrscheinlichkeit auf das Vorliegen einer SARS-CoV-2-Infektion hinweist. Leider wird dieses Symptom in den Meldedaten nicht als einzelnes Symptom übermittelt. Dieser Befund zu Kopfschmerz und SARS-Cov‑2 bei Kindern im Kita-Alter sollte in epidemiologischen und klinischen Studien weiter untersucht werden. Studien mit prospektiver, täglicher Erfassung von Symptomen bei nichthospitalisierten Kindern im Kita-Alter sind aufwendig. Insbesondere die Rekrutierung ist schwierig. In der Pandemie hat sich gezeigt, dass betroffene Kinder oftmals nicht beim Kinderarzt/bei der Kinderärztin vorstellig werden. Detaillierte, zuverlässige Daten zu nichthospitalisierten Kindern sind daher eher im Rahmen kleinerer Studien mit kleineren Stichproben, wie der COALA-Studie, eine wichtige Ergänzung zu Meldedaten. Die Meldedaten bieten hingegen über die Vollerfassung aller diagnostizierten Fälle eine sehr aussagekräftige und fundierte Datenbasis.

Eine COVID-19-Impfung ist in Deutschland zum aktuellen Zeitpunkt für Kinder ab 5 Jahren empfohlen [7]. Ob eine Impfung auch für jüngere Kinder (bis einschließlich 4 Jahre) sinnvoll sein könnte, kann aus der vorliegenden Studie nicht abgeleitet werden und muss in Zusammenschau mit u. a. Studienergebnissen zur Symptomatik und deren Schwere bei (Re‑)Infektionen durch neue Virusvarianten, Durchbruchsinfektionen und Langzeitsymptomen nach einer SARS-CoV-2-Infektion (Long-COVID) und deren Behandelbarkeit beurteilt werden. Auch zur Symptomatik bei Durchbruchsinfektionen nach einer COVID-19-Infektion liegen unseres Wissens keine kinderspezifischen Studienergebnisse vor. Daten zu Erwachsenen zeigten bei Durchbruchsinfektionen mit der Delta-Variante einen Vorteil hinsichtlich der Vermeidung schwerer Symptome im Vergleich zu ungeimpften Personen [6]. Inwiefern sich dies auf Kinder und z. B. die Omikron-Variante übertragen lässt, sollte weiter untersucht werden.

## Schlussfolgerung

Die Zusammenschau der Erhebungen aus COALA mit den Meldedaten bietet einen deutlichen Mehrwert. Schnupfen gehört zu den häufigsten COVID-19-Symptomen bei infizierten Kindern, ist aber wenig spezifisch. Die Daten belegen, dass ein hoher Anteil an mit SARS-CoV‑2 infizierten Kita-Kindern keine Symptome aufweist, und deuten darauf hin, dass Kita-Kinder häufiger einen milden bis sehr milden Krankheitsverlauf ohne „schwerere“ Symptome, wie Fieber, haben als aus den Meldedaten ersichtlich.

## Supplementary Information




