# Supplementary material for: Symptoms of SARS-CoV-2 infections in children of day care age
Source: Monatsschr Kinderheilkd. 2022 Nov 9;170(12):1113–21. [Article in German] doi: 10.1007/s00112-022-01640-3 (PMC9645339; doi:10.1007/s00112-022-01640-3)
Supplement: Supplementary file 1 [file 112_2022_1640_MOESM1_ESM.docx]

Tabelle 4: Anzahl und Anteil der Nennungen von Symptomen bei symptomatischen SARS-CoV-2-Fällen von Kindern im Alter von 1-6 Jahren; Vergleich der COALA-Studie (n=25) und der Meldedaten (n=54.382).

| **Symptom** | **Ausbruchsuntersuchungen^1^**  Fallzahl (n)  Anteil (in Prozent) | **Meldedaten**  Fallzahl (n)  Anteil (in Prozent) |
| --- | --- | --- |
| **Mindestens ein Symptom** | 25  100% | 54.382  100% |
| **Schnupfen** | 14  56% | 22.195  41% |
| **Kopfschmerzen** | 6  24% | x |
| **Halsschmerzen** | 6  24% | 6.427  12% |
| **Fieber** | 6  24% | 22.931  42% |
| **Husten** | 5  20% | 22.236  41% |
| **Durchfall** | 4  16% | 3.180  6% |
| **Gliederschmerzen** | 3  12% | x |
| **Geschmacksstörung/**  **Geschmacksverlust** | 1  4% | 958  2% |
| **Geruchsstörung/**  **Geruchsverlust** | 1  4% | 718  1% |
| **Übelkeit** | 1  4% | x |
| **Schüttelfrost** | 0  0% | x |
| **Atemnot** | 1  4% | 552  1% |
| **Atemschmerzen** | 0  0% | x |
| **Allgemeine Krankheitszeichen** | x | 18.196  33% |
| **Sonstige*** | –  – | 264  0,5% |

^1^: Symptomnennung unter positiven Kindern; x = Diese Symptome wurden so nicht erfasst. *: Im Meldesystem konnten außerdem Pneumonie: 78 (0,1%), ARDS: 93 (0,2%), Beatmung: 9 (<0,0%), Tachykardie: 27 (<0,0%) und Tachypnoe: 57 (0,1%) erfasst werden, die hier als „Sonstige“ zusammengefasst wurden.
